# Occupational Health Challenges for Aviation Workers Amid the Changing Climate: A Narrative Review

**DOI:** 10.7759/cureus.55935

**Published:** 2024-03-11

**Authors:** Piercarlo Minoretti, Manuel Gómez Serrano, Miryam Liaño Riera, Andrés Santiago Sáez, Ángel García Martín

**Affiliations:** 1 Occupational Health, Studio Minoretti, Oggiono, ITA; 2 Legal Medicine, Psychiatry and Pathology, Complutense University of Madrid, Madrid, ESP; 3 Legal Medicine, Hospital Clinico San Carlos, Madrid, ESP

**Keywords:** level of resilience, occupational health hazards, flight, aviation, climate change and its effect on life and health

## Abstract

Although there are many forecasts regarding the impact of climate change on the aviation sector, a critical but frequently neglected dimension is the occupational safety risks faced by aviation professionals. This narrative review explores the potential impacts of the changing climate on the health and safety of aviation personnel. Furthermore, we examine the significance of resilience in helping these workers adapt and effectively manage climate-related challenges in their professional lives. Climate change poses increasing threats to the well-being of flight personnel through elevated temperatures, heightened ultraviolet radiation exposure, increased mental workload from extreme weather events, and other psychological stressors. Building resilience through workforce training, planning, and adaptation can reduce vulnerability. In future research, the iterative process of selecting measurement components to gauge the impact of climate change should balance feasibility, relevance for stakeholders, and accurately capturing exposure effects. For instance, while salivary cortisol measures stress biologically, assessments of depression or burnout may provide more nuanced insights on pilot health for industry decision-makers managing climate impacts. In conclusion, a strategic emphasis on enhancing the physical and psychological well-being of the aviation workforce is imperative for facilitating a more efficient adaptation within the sector. This is of paramount importance, considering the critical function that aviation serves in fostering human connectivity. Consequently, it is essential for regulatory bodies and policymakers to prioritize the safeguarding of employee health in the face of climate change challenges.

## Introduction and background

Climate change, defined by consistent shifts in temperature and weather patterns, is predominantly driven by anthropogenic emissions of greenhouse gases (GHG), notably carbon dioxide (CO_2_) [1]. The unfolding crisis poses grave dangers to global stability, with disruptive impacts across social structures, economic systems, biodiversity, and human health [2]. Failure to mitigate climate change currently ranks among the top five gravest dangers for the coming decade, along with intensifying extreme weather events, biodiversity loss and ecosystem collapse, crises sparked by dwindling natural resources, and environmental degradation from human activities [3]. The need to limit global warming is thus becoming increasingly urgent. Multiple international organizations have called for substantial and immediate reductions in emissions, along with adaptation measures, to secure a sustainable, habitable future [4]. However, the window of opportunity to meet the 1.5°C target is rapidly closing, further sharpening an already perilous situation [5].

**Figure 1:**
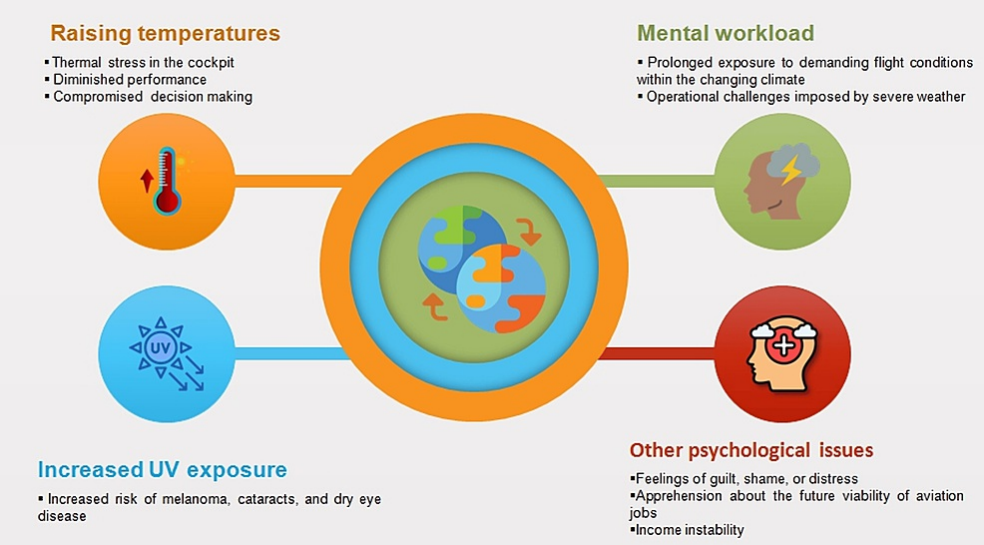
Summary of the main occupational health challenges faced by aviation workers in a changing climate Image credits: Piercarlo Minoretti

The aviation industry and climate change have a complex, interdependent relationship. On the one hand, aviation substantially contributes to anthropogenic climate-driven impacts. Current estimates indicate that the sector directly accounts for approximately 2.5-3% of global GHG emissions from fossil fuel combustion and industrial processes [6]. Moreover, when factoring in non-CO_2_ impacts like contrails and nitrogen oxide emissions at altitude, aviation’s total climate impact is estimated to be at least 3.5% of human-caused warming [7]. Despite the sector's heavy dependence on fossil fuels and the technical difficulties of transitioning to sustainable alternatives, mounting pressure exists to mitigate aviation carbon emissions [8]. On the other hand, this industry remains highly vulnerable to the disruptive impacts of climate change, including operational interruptions, infrastructure damage, airport closures, and flight cancellations from shifts in temperature and weather patterns [9]. While numerous predictions exist regarding how shifts in temperature and weather patterns will impact the sector, one commonly overlooked area is the occupational safety hazards posed to aviation workers. However, climate change is expected to increasingly endanger the health of flight personnel due to higher temperatures, increased ultraviolet (UV) radiation exposure, greater mental workload from managing aviation operations during extreme weather events, and other forms of psychological stress (Figure 1).

In this narrative review, we explore the potential impacts of climate change on the health and safety of professionals within the aviation industry. Additionally, we delve into the significance of resilience in assisting these workers to adapt and effectively manage the challenges and obstacles posed by climate-related factors in their line of work.

## Review

Raising temperatures

Climate change is projected to raise global temperatures over the next few decades [2]. This warming trend poses occupational health risks to pilots, who may experience excessive cockpit heat during flights [10]. These effects may be compounded by high humidity levels that impede the body’s ability to cool through sweating [10]. High cockpit temperatures and humidity can cause pilots discomfort, dehydration, fatigue, and potentially more serious complications (e.g., heat stroke and heat cramps) [11-14]. As a result, pilots may suffer declines in cognitive performances, including vigilance, reaction time, and decision-making, which are critical for flight safety [10,11]. Among aviation personnel, military [10] and helicopter [11,12,15] pilots appear especially vulnerable to heat strain due to frequent exposure to extreme conditions. For example, fighter pilots routinely experience intense heat and humidity during pre-flight duties, low-altitude missions, and combat readiness tasks [10]. Cockpit temperatures can exceed 45°C under such conditions, which are expected to intensify with climate change [10]. Notably, the use of anti-G suits, which are essential for counteracting high gravitational forces, may further increase the thermal load in this professional category [10]. This is attributed to the inflatable counter-pressure vest designed to facilitate positive pressure breathing, which, while crucial for maintaining G-tolerance, exacerbates the heat stress experienced during flight [16]. Helicopter pilots represent another occupational group that is distinctly vulnerable to heat stress due to the unique conditions within their work environment [11,12,15]. In particular, the cockpit’s glass canopy design can exacerbate heat load, most notably in regions with high ambient temperatures, potentially compromising pilot performance [11]. This issue becomes especially pronounced during ground standby operations, where the lack of airflow that would normally occur during flight allows cockpit temperatures to rise approximately 20°C above the surrounding environment [11,12,15]. Mirroring findings among military pilots [10], research indicates that helicopter pilots suffering from heat stress may experience diminished performance and compromised decision-making abilities [11]. Furthermore, a direct correlation exists between thermal stress within the cockpit and an increased frequency of errors committed by pilots, indicating a dose-response relationship [11]. To mitigate excessive cockpit heat and safeguard pilot health and well-being, several key strategies can be employed. Theoretically, optimizing operational procedures - such as scheduling flights during the cooler parts of the day, minimizing ground time to reduce heat exposure, adjusting departure times to circumvent peak heat periods, and ensuring crew rest in adequately cool environments - could prove beneficial. However, the practical application of many of these adjustments faces significant real-world constraints. Consequently, we believe that crew training becomes necessary, with an emphasis on recognizing the signs and symptoms of heat stress, implementing preventive measures against heat stress, and fostering awareness about heat stress. Adopting these strategies could, at least in part, mitigate the risks associated with excessive cockpit heat exposure, thereby enhancing pilot health, comfort, and performance.

Increased ultraviolet radiation exposure

Airline pilots may be exposed to increased levels of UV-A radiation (wavelength: 315-400 nm) through airplane windshields during flight [17], and one study found that the UV exposure during a 56.6-min flight at 30,000 feet was equivalent to 20-30 min in a tanning bed [18]. Although some authors were unable to detect UV-A or UV-B radiation (wavelength: 280-315 nm) in airplane cabins or cockpits [19], there is increasing evidence that pilots carry genomic signatures consistent with UV-induced DNA damage [20,21]. This observation is further strengthened by occupational health studies showing that this professional category is at increased risk of UV-related disorders, including skin cancer [22,23], nuclear cataract [24], and dry eye disease [25]. Remarkably, a meta-analysis by Sanlorenzo et al. [23] revealed that pilots have approximately twice the incidence of malignant melanoma compared to the general population. Additionally, pilots had an approximately 83% higher melanoma mortality rate compared to the general population [23]. A separate study found that the odds ratio for the development of nuclear cataract was 3.02 for pilots compared with non-pilots after adjustment for age, smoking status, and sunbathing habits [24]. This indicates that even when accounting for potential confounding factors, pilots had a three times higher risk of developing nuclear cataracts compared to non-pilots. UV radiation exposure can also result in increased oxidative stress and inflammation on the ocular surface, which are underlying mechanisms in the pathogenesis of dry eye disease [26]. This condition often manifests symptomatically with feelings of irritation, grittiness, or visual disturbances but can also occur asymptomatically, including among aircrew personnel [25]. In the context of climate change, the health hazards posed by UV radiation are becoming a more pressing issue for aviation workers due to the thinning of the stratospheric ozone layer [27]. This depletion is expected to heighten the already significant UV exposure risks for crew members at high altitudes. Additionally, during long-haul flights, climate change-related phenomena, such as heatwaves and changes in cloud cover, water vapor, and aerosols [27], could further influence UV exposure on a regional scale. Hence, we believe that it is necessary for the aviation industry to adopt protective measures for its employees due to the potential increase in UV exposure. One key mitigation strategy we advocate for is the enhancement of UV protection in aircraft windshields, which currently exhibit inconsistent UV-blocking capabilities. Additionally, we recommend other protective actions, such as promoting the use of sunscreen, encouraging the wearing of sunglasses, and advocating for the use of protective clothing, to shield aviation workers from the immediate and cumulative effects of UV exposure. These initiatives have the potential to address the convergence of climate change, elevated UV exposure at altitude, and the well-being of aviation personnel.

Mental workload and stress associated with extreme weather conditions

Weather conditions directly or indirectly contribute to nearly 25% of all aviation accidents, playing a major role in aviation safety [28]. Pilots and other aviation personnel frequently experience substantial psychological stress when navigating severe weather, which can potentially impair decision-making and situational awareness. The increasing frequency and intensity of extreme weather events present escalating safety challenges and demanding flight conditions [29,30]. In the face of climate change, pilots may be required to manage a multitude of stressors, such as severe turbulence, thunderstorms, icing, anxious passengers, sleep deprivation, and unpredictable schedules. Prolonged exposure without adequate recovery can increase anxiety, depression, and sleep disorders. The temperature rise also impacts aircraft performance from less dense air, particularly during takeoff. On hot and humid days, aircrafts require more time and distance to accelerate, achieve lift, and ascend, which not only heightens the risk of accidents but also intensifies the demands on aviation personnel. In extreme heat, airplanes may need to reduce their weight by 10-30% to compensate [31], which can increase the workload for aviation personnel and potentially lead to more accidents. Climate change is also projected to increase the frequency and intensity of thunderstorms and lightning activity, with estimates suggesting a 12% increase in lightning for every 1°C rise in temperature [32]. Additionally, clear-air turbulence (CAT), which occurs without visual cues and is difficult to predict [33], is becoming more severe due to climate change. CATs are at least in part caused by increased wind shear in the jet streams from warmer air resulting from CO_2_ emissions [34]. The unpredictable nature of this phenomenon can elevate stress levels for pilots, who must maintain high alertness and make critical decisions under such conditions. This can exacerbate mental health issues, contributing to anxiety and other psychological symptoms. Mental health issues significantly influence determinations of a pilot’s fitness for duty, with psychiatric and psychological disorders being primary reasons for grounding pilots [35,36]. The growing operational burden imposed by climate change has the potential to exacerbate these mental health challenges, potentially leading to heightened anxiety, stress, and burnout. This is a pressing concern, given the vital role of psychological fitness in pilot performance and the overall safety of air travel [37].

Other psychological issues

The psychological toll of climate change on aviation personnel can be complex, stemming from a plethora of occupational and financial pressures. As a major contributor to GHG emissions [7-9], the aviation industry may elicit feelings of guilt, shame, or distress among its workforce regarding its environmental impact. Encounters with climate activism can further exacerbate these negative emotions. Apprehension about the future viability of aviation jobs given tighter environmental regulations can also take a psychological toll, as can anxiety related to income instability from grounded flights, rising operating expenses, and potential job losses. Without proper support, these mounting stressors can elevate the risk for mental health conditions like anxiety, depression, and burnout among aviation staff. To safeguard the well-being of this critical workforce amid ongoing environmental and industry transformations, providing accessible mental health education, screenings, counseling, and treatment is imperative [38,39]. Such proactive measures can help aviation personnel constructively cope with climate-related emotional burdens.

Discussion

As climate change leads to more frequent extreme weather events and poses increasing operational challenges, aviation personnel will need to implement resilience strategies to protect both their physical and mental health. The concept of resilience, traditionally defined as the ability to recover from adversity, is increasingly recognized in the medical literature. Initially referring to the elastic properties of materials, the term’s usage has expanded to describe resilient systems, communities, and individuals [40]. In recent years, substantial research has portrayed psychological resilience, highlighting adaptive attitudes and behaviors that promote well-being and potential growth after stressful events [41]. Furthermore, the distinct yet related concept of physical resilience has emerged, pertaining to the capability to regain or enhance functionality amid physical impairments or diseases [42]. We propose that climate change resilience [43] is fundamental for maintaining long-term health in aviation workers. This construct must consider both exogenous factors, such as rising temperatures and UV radiation exposure, and endogenous processes, including mental health issues stemming from operational challenges as well as occupational and economic pressures. Identifying individual resilience factors will be crucial to elucidate the overall impact of climate change on the aviation workforce. For instance, UV radiation resistance may vary due to genetic differences influencing DNA repair mechanisms [44], and evidence shows that low baseline DNA damage predicts resilience to radiation [45]. Strategies to enhance climate change resilience could include risk assessment, scenario planning, and adaptive management. We posit that refining the measurement of climate change resilience among diverse aviation worker cohorts may yield novel insights and intervention targets. This should involve constructing questionnaires to probe psychosocial factors and conducting in-depth evaluations of essential functions in major physiological systems, including the neurological, cardiovascular, immunological, and musculoskeletal systems.

## Conclusions

Regulators and decision-makers in aviation should pay adequate attention to protecting workers’ health in the face of climate change. Building resilience against climate impacts through workforce training, planning, and adaptation can reduce worker stress and vulnerability. Future research should also identify appropriate metrics to measure the health effects of climate change on aviation workers. Specifically, studies could formulate targeted research questions on the impacts of specific climate exposures (e.g., heat waves) on outcomes like pilots’ cognitive performance. The iterative process of selecting measurement components should balance feasibility, relevance for stakeholders, and accurately capturing exposure effects. For instance, while salivary cortisol measures stress biologically, assessments of depression or burnout may provide more nuanced insights on pilot health for industry decision-makers working to manage climate impacts. Overall, a strict focus on supporting the workforce's physical and psychological health will enable more effective aviation sector adaptation.
